# Modeling Magnification and Anisotropy in the Primate Foveal Confluence

**DOI:** 10.1371/journal.pcbi.1000651

**Published:** 2010-01-29

**Authors:** Mark M. Schira, Christopher W. Tyler, Branka Spehar, Michael Breakspear

**Affiliations:** 1 School of Psychiatry and The Black Dog Institute, University of New South Wales, Sydney Australia; 2 School of Psychology, University of New South Wales, Sydney, Australia; 3 The Smith-Kettlewell Eye Research Institute, San Francisco, California, United States of America; 4 Queensland Institute of Medical Research and the Royal Brisbane and Woman's Hospital, Queensland, Australia; Universitat Pompeu Fabra, Spain

## Abstract

A basic organizational principle of the primate visual system is that it maps the visual environment repeatedly and retinotopically onto cortex. Simple algebraic models can be used to describe the projection from visual space to cortical space not only for V1, but also for the complex of areas V1, V2 and V3. Typically a conformal (angle-preserving) projection ensuring local isotropy is regarded as ideal and primate visual cortex is often regarded as an approximation of this ideal. However, empirical data show systematic deviations from this ideal that are especially relevant in the foveal projection. The aims of this study were to map the nature of anisotropy predicted by existing models, to investigate the optimization targets faced by different types of retino-cortical maps, and finally to propose a novel map that better models empirical data than other candidates. The retino-cortical map can be optimized towards a space-conserving homogenous representation or a quasi-conformal mapping. The latter would require a significantly enlarged representation of specific parts of the cortical maps. In particular it would require significant enlargement of parafoveal V2 and V3 which is not supported by empirical data. Further, the recently published principal layout of the foveal singularity cannot be explained by existing models. We suggest a new model that accurately describes foveal data, minimizing cortical surface area in the periphery but suggesting that local isotropy dominates the most foveal part at the expense of additional cortical surface. The foveal confluence is an important example of the detailed trade-offs between the compromises required for the mapping of environmental space to a complex of neighboring cortical areas. Our models demonstrate that the organization follows clear morphogenetic principles that are essential for our understanding of foveal vision in daily life.

## Author Summary

Cortical areas V1, V2 and V3 each contain a complete and orderly representation of the visual environment, and their detailed layout is often studied. Here we discuss and compare algebraic mapping functions providing a transformation from visual field to cortical maps. Such algebraic models allow prediction of the layout in individuals from sparse data and advance our understanding of the mapping principles guiding the cortical organization in this important brain region. We show the deficiencies in previous work and provide the first plausible account of the combined layout of the first three visual maps, deriving an optimal compromise between the desirable principles of local isotropy of the maps and the extent of cortex that they occupy. This formal mapping scheme has only a few parameters but accurately predicts the layout of cortical areas, particularly the confluence of the three retinotopic areas in the central fovea, which we have only recently resolved. This scheme highlights the result that V2 and V3 have larger cortical representations than V1 in the central fovea, explaining the release from acuity crowding in this region. In summary, our novel model describes the high-resolution retinotopic mapping data to the level of their existing empirical accuracy.

## Introduction

Primate visual cortex contains several seemingly-complete topographic representations of the visual field. The three major representations V1, V2 and V3 are considered to be homologous among primates and are well studied in a large number of species. In 1969 Zeki [1] reported that the foveal parts of the V1, V2 and V3 maps all converge towards a common center, not unlike pie wedges meeting at the center of the pie. This arrangement was later termed ‘the foveal confluence’. Since then the general layout of early visual areas has been confirmed and extended in various primate species, but their precise layout in the fovea remained unknown.

Algebraic forms of the retinocortical map are important for a number of reasons. Firstly, they allow explicit formulation of key properties, such as the relationship between cortical magnification and eccentricity. Secondly, they allow one to understand whether different forms of distortion are necessary and, if so, their inter-relationships. Thirdly, they can be applied in empirical studies to predict complete visual field maps based on sparsely acquired data. And finally, they provide a means of comparing quantitative predictions across different analysis techniques, such as neurophysiology, brain imaging and psychophysics. Over the past decades, a number of such candidate models have appeared. However, existing retinocortical models are predicated on a “pie wedge” organization of V1, V2 and V3 [2] and are incompatible with recent, high resolution fMRI data [3] that instead reveal a banded architecture for the V2 and V3 maps.

We start by reviewing the conceptual and computational principles that form the basis of characterizing the retino-cortical projection and their relationship to recent empirical data. These concepts then form the basis of our subsequent evaluation of existing models, and finally lead to a new model which is the first to incorporate the recently-observed banded architecture at the fovea.

### Magnification and Anisotropy

A key feature of these ‘first tier’ retinotopic areas is that the representation of the center of the visual field, the fovea, is greatly enlarged. The extent of this enlargement is often measured and termed magnification (*M*). Magnification is commonly estimated [4],[5],[6],[7],[8],[9],[10],[11],[12],[13],[14],[15],[16] and in its simplest form is often described by the function:
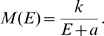
(1)In this notation, *M* is typically measured in mm on cortex per deg in the visual field, and *M* depends on eccentricity (*E*) but is invariant with angular (‘polar’) position (*P*). This latter assumption is a valid first order approximation [5],[13],[15],[16],[17],[18].

For a more complete understanding, however, the validity of the assumption of polar-angle invariance has to be considered, *i.e.*, magnification may depend not only on eccentricity, but also on polar position. Further, magnification itself can be viewed either as a linear property (*i.e.*, mm of cortex/deg of visual field) or as an areal measure (*M_a_* = mm^2^ cortex/deg^2^ of the visual field). While the areal measure is by definition direction-independent, linear magnification may be direction dependent for each location on cortex and is typically described along isoeccentricity lines (typically designated as *M_e_(E,P)*) and along isopolar lines (*M_p_ (E,P)*). There is a simple relation between these magnifications with M_a_ = M_e_*M_p_. Now, *M_p_* and *M_e_* may actually be different at a given part of cortex: this is usually termed aniostropy. Here we define 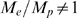 as ***local anisotropy*** (or *local isotropy* if *M_p_*/*M_e_* = 1), which essentially means that a square or circle in the visual field is represented as elongated in one direction on cortex. Variations in M across the cortex are termed ***meridional anisotropies***. These concepts are essential for understanding this paper and are discussed in detail in Schira et al. 2007 [15].

### Candidate Analytic Models for V1

**Figure 1:**
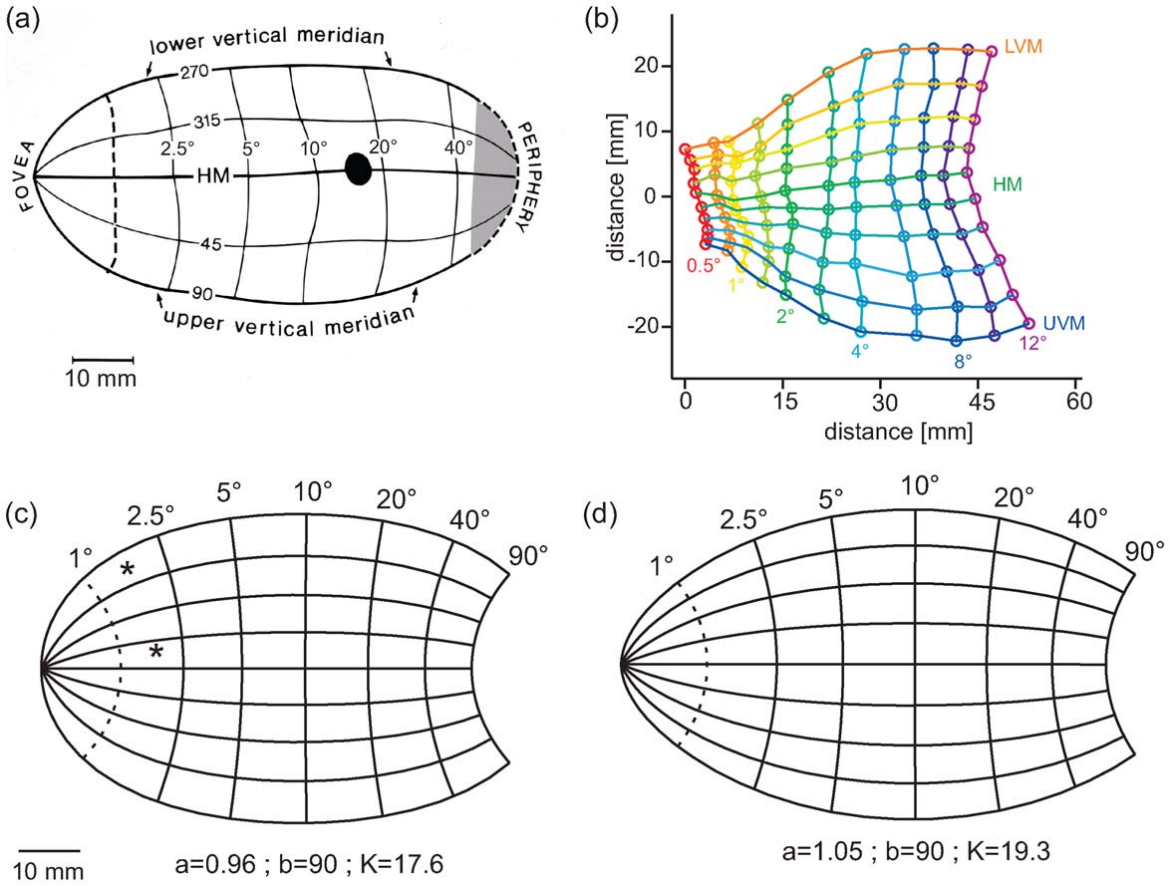
Fully two-dimensional maps of human V1. (a) Schematic of the human visual field as suggested by Horton and Hoyt [29], based on the visual field loss of 3 patients and the assumption of equivalence with monkey data (adapted version provided by Horton, personal communication). (b) Quantitative, 2-D reconstruction of human V1 based on fMRI recordings on 8 healthy subject [15]. (c) The Dipole model using the classical log-polar transform as proposed by Schwartz [19], parameters based on optimal fit to the data in (b). Note that the areas marked by * represent analogous portions of visual field, varying only by polar position and not in eccentricity. According to equation 1 (and a broad range of data) *M_a_* should be constant. However, the classical log-polar transform predicts a significant change of *M_a_* with polar position (by a factor of 1.7 for this particular set of parameters) - in contradiction to eq. 1. (d) The dipole versions of the Double-Sech model, parameters chosen for optimal fit to the data in (b). The Double-Sech model predicts no change in *M_a_* with polar position.

In 1977, Schwartz [19] proposed an analytical expression to describe the two-dimensional mapping between retinal and cortical coordinates by the use of the complex variable *z* to represent both retinal eccentricity (*E*) in the real part and angular deviation from the horizontal meridian (*P*) simultaneously in the imaginary part. He suggested that the map of visual space in area V1, symbolized by the function *w*, could be approximated by a complex-log transform of the retinal image:
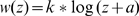
(2)where *a* defines the limit of the foveal singularity, *k* is a scaling constant and 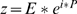. This straightforward model provides a surprisingly good approximation of the mapping principles of primate V1, at least for the central part of the visual field. Since this splitting the model in two halves is inconvenient for this manuscript we will use a more explicit version of the Schwartz model integrating *z*:
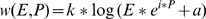
(3)However, since this does not predict the diminution of the visual cortex at the periphery, accordingly to incorporate the tapering of visual cortex not only towards the fovea (later termed Monopole model), but also for the periphery the initial model was extended with a second parameter *b*,
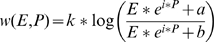
(4)resulting in the Dipole model [20] illustrated in Figure 1c.

**Figure 2:**
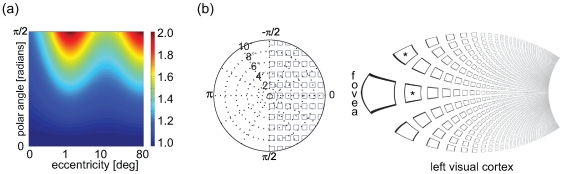
Anisotropy resulting from the Double-Sech mapping function. (a) The shear function introduced by the Double-Sech model (Eq. 6) plotted as a joint function of eccentricity and polar angle. The shear changes with both variables and is generally stronger at the vertical meridian. (b) The resulting anisotropy demonstrated by the mapping of a set of squares in the right visual field to the left hemisphere V1. The two marked squares demonstrate the effect of the predicted anisotropy; both squares are equal in size (area), but the square projected on the vertical meridian is elongated.

Both Monopole and Dipole models propose a change of *M_a_* with polar position (see Figure 1c). At the time these models were proposed there were no data sufficiently precise available to test their predictions, but early on Sakitt 1982 [21] argued that macaque V1 could not be isotropic based on geometrical considerations. Since then, various detailed estimates of magnification suggest that for V1, *M_a_* is in fact constant for a given eccentricity (Gattass et al. 1987 [16] for cebus monkey, Tootell et al 1988 [17] for macaque monkey, Adams and Horton 2003 [13] for squirrel monkey, Schira et al. 2007 [15] for human), an observation that is not consistent with the isotropic Schwartz model. Accordingly, Schira et al. [15] proposed a modification of the classical log-polar transform, the Double-Sech model, introducing a shear function 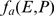 the monopole model, equation 3,
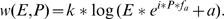
(5)This shear function is described in Figure 2a and was originally determined iteratively to a constant *M_a_* with polar position as illustrated in Figure 2b.

For simplicity we use an algebraic form to approximate this function:
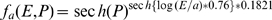
(6)


For a dipole version of the Double-Sech model, a second shear 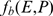 is implemented for the dipole into the appropriate place in the denominator, hence
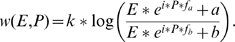
(7)It is important to note that, despite the complex equation approximating the shear function, this modification does not add any additional free parameters to the model, but merely incorporates a different geometric principle with no additional degrees of freedom. As for the classical Log-Polar transform, the full Double-Sech model has 2 structural parameters *a* and *b*, plus a single size-scaling parameter *k*.

It is important to note that, as a result of the Double-Sech shear function (as *any* shear function), the model is not longer conformal, especially at the vertical meridians close to eccentricities of a = 1.05° and b = 90°, however the shape of the resulting model is more elongated, narrower than the shape predicted by the original Schwartz model.

### The V1-V2-V3 complex

**Figure 3:**
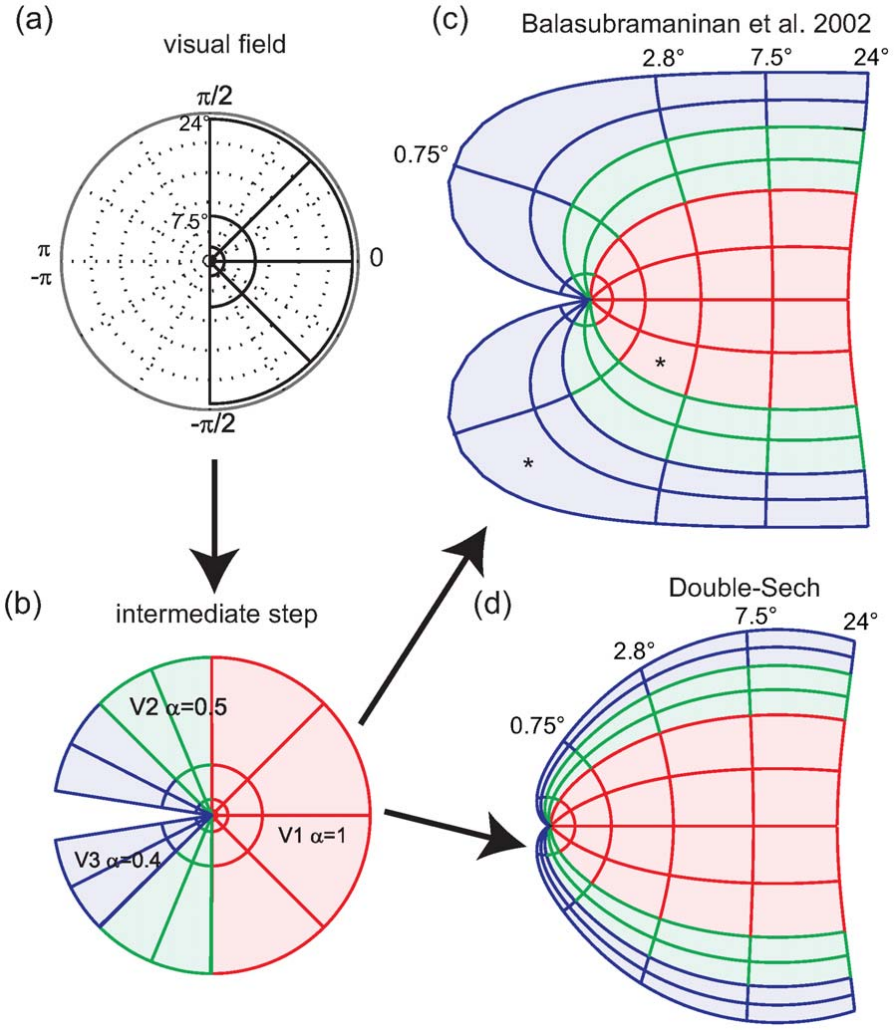
Modeling the V1–V2–V3 complex. (a) The visual field, i.e. the starting point of the model. (b) In a first step as suggested by Balasubramanian et al. (2002) [2], the visual field is transformed by simple reflections into an intermediate space, which, in a second step (c), is projected to cortical space using the classical log-polar function. Note that projected size increases dramatically near the foveal projection with increasing polar position. This effect is maximal for eccentricity ranges around *a* = 1.05°. The two areas marked with a star represent the same part of the visual field, but the representation is significantly larger in V3 (blue) than in V1 (red). This expansion is not supported by reported data [15],[27] (d) The Double-Sech model corrects this undesirable behavior. V2 and V3 maintain a constant size ratio to V1 throughout all eccentricities. In the Double-Sech model, the surface area relation between V1–V3 is simply specified by the ratio of the *α_1_*–*α_3_* parameters. In the example illustrated here, *α_3_* = 0.4 for V3 while *α_1_* = 1 for V1. Accordingly, the area ratio is V3∶V1 is 0.4∶1.

In 2002, Balasubramanian et al. [2] introduced a concept for extending the original model (the dipole variant ) allowing to model not only V1 but a complex of the areas V1, V2 and V3. The central idea of this ‘wedge dipole’ concept is a two-step procedure. First, the visual hemifield is split into two quadrants that are mirrored along the upper and lower vertical meridians to form V2v and V2d - very similar to the situation observed on visual cortex. V3 is then formed by iterative mirroring of the quadrants. This mirroring procedure is illustrated in Figure 3b, with the visual areas color-coded. In the second step this compound map is then transformed via the classical log-polar transform, resulting in an extended cortical map of the V1-3 complex as presented in Figure 3c. Unfortunately, the main problem of the classical log-polar transform – namely the predicted increase *M_a_* with increasing polar position - becomes dramatically more severe for this model. The foveal projection balloons out to a marked extent at the eccentricity of parameter *a* (here assumed to be 1.05°). However, as evident in Figure 3d, we were able to remedy this problem by passing the sheared V1-3 complex through the Double-Sech model instead.

## Results

Analytic two-dimensional mapping functions provide the opportunity to test a multitude of properties, and finally compare key properties with real data. We hence undertook a parametric survey of existing retino-cortical models, focusing on various types and magnitudes of anisotropy. These anisotropies are characterized first in the existing candidate models, the log-polar transform and the Double-Sech model. We then re-examine the nature of these distortions and then suggest an adjustment of the model which incorporates the banded nature of V2 and V3.

### Meridional Anisotropy

As introduced in Section 1, magnification is usually estimated with respect to eccentricity and is assumed to be independent of polar angle, *i.e. M_a_*(*E,P*_1_) = *M_a_*(*E,P*_2_) = *M_a_(E)*. We now investigate ratios of areal magnification suggested by the candidate models, as magnification varies with angular position: *M_a_(E′,P)* for fixed *E = E′*. Different values for *M_a_* (for example on the horizontal meridian *M_a_*(*E′*,0) and the vertical meridian *M_a_*(*E′*,π/2) ), would signify a deviation from the simple rule that magnification depends on eccentricity but not on polar position. We refer to this dependence on polar position as ***meridional anisotropy*** (also termed radial bias [22]).

We further extend the analysis across visual areas, normalizing the magnification in V2 and V3 to the magnification in V1 for the horizontal meridian. In general empirical data suggest that V2 and V3 are both smaller than V1, accordingly the *meridional anisotropy* values in V2 and V3 should be less than 1.

**Figure 4 pcbi-1000651-g004:**
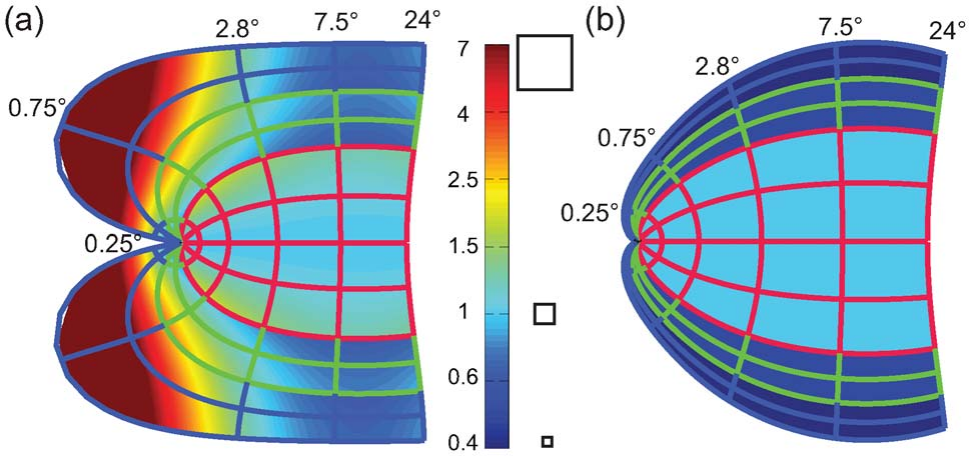
Meridional anisotropy. Values smaller than 1 (dark bluish colors) indicate that magnification is smaller compared to the horizontal meridian in V1. Values larger than 1 (reddish colors) indicate a larger magnification. (a) The Log-Polar model suggests large amounts of meridional anisotropy. In particular it suggests up to 7 times the magnification for V3 as for V1. (b). The Double-Sech model predicts rather simple patterns of meridional anisotropy. Essentially, it predicts a constant anisotropy for each retinotopic area (*i.e.* no change of meridional anisotropy within an area). The amount of anisotropy for a given area is simply determined by the α parameter. V3 has a meridional anisotropy of 0.4 (determined by α_3_ = 0.4).

Figure 4 depicts the ratio of *M_a_* at a given part of the projection to *M_a_* at the same eccentricity on the horizontal meridian of V1. Figure 4a shows the results for the classical Log-Polar transformation, which exhibits a strong amount of meridional anisotropy, in particular for eccentricity ranges from 0.4–2°. In comparison, Figure 4b shows the result for the Double-Sech model, exhibiting virtually no meridional anisotropy.

Empirical results for meridional anisotropy in primates are at best mixed, but certainly not in the direction or of the magnitude predicted by the classic Log-Polar transform. In the macaque monkey, some degree of meridional anisotropy was reported [22] – although in the opposite direction to that predicted by the classical Log-Polar transform. However, results from a later report [17] were not consistent with this finding, at least for the central part of the visual field. In squirrel monkey V1, Adams & Horton, 2003 found no evidence for significant meridional anisotropy [13], likewise in Cebus monkey [16] and humans [15].

### Local Anisotropy

As introduced in the Section 1, *linear* magnification can be measured parallel to isopolar lines (*M_p_*) or parallel to isoeccentricity lines (*M_e_*) and in general, *M_e_* and *M_p_* are not necessarily equal at a given point in the visual field. There are several reports of such ***local anisotropies*** [13],[15],[17],[23],[24],[25].

**Figure 5:**
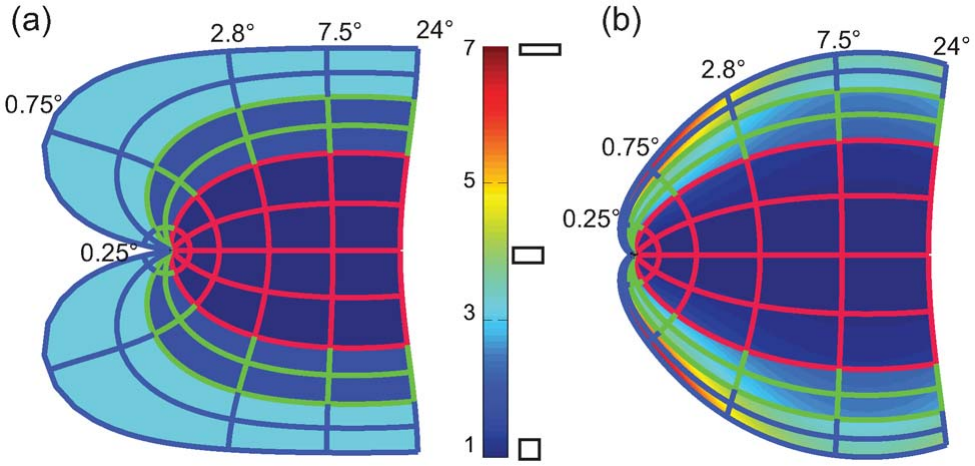
Local anistropy. (a) The classical Log-Polar transform predicts a simple pattern of local anisotropies. Here α directly determines local anisotropy, α_3_ = 0.4, resulting in a local anisotropy of 2.5 (1/α_3_). (b) The Double-Sech model predicts a more complicated pattern of local anisotropy, suggesting fairly large degrees of local anisotropy, especially near the V3 foveal projection.

Figure 5 depicts the local anisotropy predicted by the classical Log-Polar transform and our Double-Sech model. For local anisotropy the organization is essentially the converse of the situation found for meridional anisotropy. Here the classical Log-Polar transform predicts a simple pattern, where local anisotropy for each area is homogenous and determined by the parameters α_1–3_, whereas the Double-Sech model predicts a complex pattern of local anisotropy to achieve the meridional isotropy observed in the previous paragraph. Summarized, Figures 4 and 5 illustrate that either local or meridional anisotropy is necessary, and that the two forms of distortion are essentially opposite ends of a continuous spectrum.

### Comparing the Model with Foveal Data

**Figure 6:**
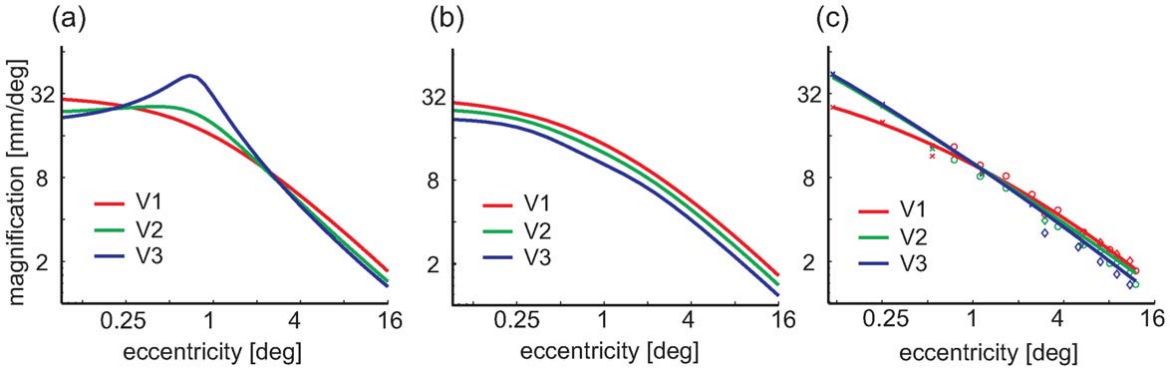
Predicted and measured areal magnification. (a) As predicted by the classical Log-Polar model, using parameters from [35] (b) The Double-Sech model, (c) Empirical data from [3] (crosses), [15] (circles) and [27] (diamonds).

Explicit linear magnification curves were estimated from both models and compared to empirical data (Figure 6). Both models result in fairly accurate predictions from 12° to 3°. However, more central than 3°, the classical Log-Polar model fails to predict the empirical data, suggesting in particular a very specific enlargement of V3 that is absent in the data. The Double-Sech model, on the other hand, predicts a constant relation of magnification between V1, V2 and V3 throughout the visual field, providing a fairly accurate prediction of magnification from 12° down to 0.75°. However, central to 0.75° the empirical data show that V3 and V2 are larger than V1, a property that is not captured by either of the present models.

**Figure 7:**
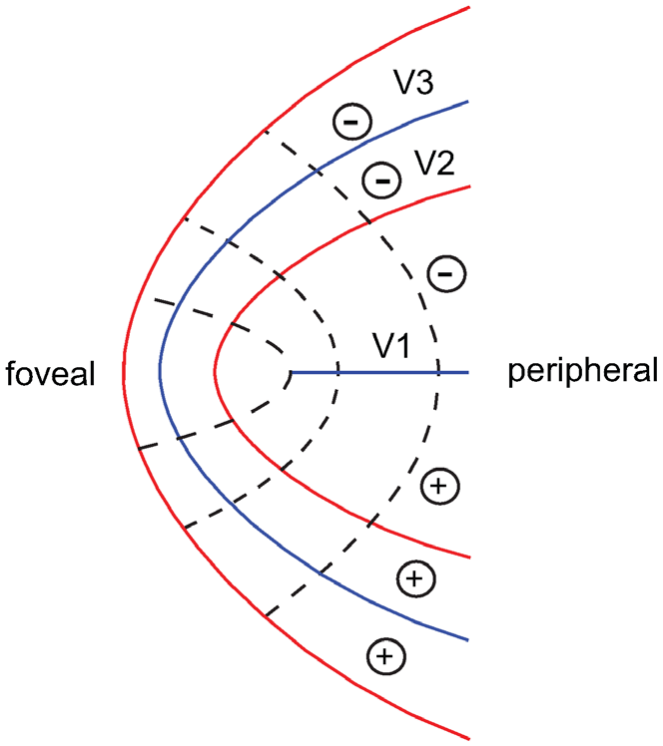
Canonical layout of the foveal confluence. This layout is derived from data [3]. Dotted lines depict isoeccentricity contours, red lines - vertical meridian and blue lines horizontal meridian representations. Empirical data suggest that V2 and V3 do not come to a point but form bands surrounding the foveal tip of V1. Plus and minus signs signify representations of the upper and lower visual field.

These discrepancies are the result of the fact that both models predict that V2 and V3 converge to a point in the centre of the fovea (see Figures 3–5), whereas measurements of the human foveal confluence [3] show that V2 and V3 form roughly parallel bands surrounding the tip of V1 (Figure 7).

### The Banded Double-Sech Model

**Figure 8:**
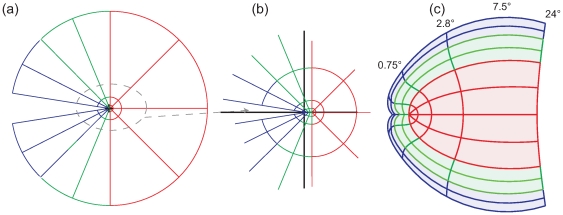
The Banded Double-Sech model. (a) The revised ‘pacman’ stage, from 0 to 24°. (b) The very central part (0–2.5°) of the visual field is enlarged to show the vertical axis shift. As can be seen, only a small shift is required to achieve the necessary trapezoidal shape for V2 and V3. (c) The resulting projection on visual cortex. V2 and V3 no longer come to a point, but rather form bands surrounding the foveal tip of V1. This banded structure has been reported for several primate species [26],[44], and also for humans [3].

Informed by the principled difference in the organization of the foveal singularity between models and data, we propose a new model, the ***Banded Double-Sech*** model of the V1-3 mapping structure. The Banded Double-Sech model introduces a critical alteration to the first step proposed by Balasubramanian [2] (Figure 2b). The basic idea is to incorporate the banded structure at the level of intermediate step – which we call the “pacman” - by transforming the V2 or V3 quadrants from triangular wedges into trapezoids and hence extend the foveal point into a line (Figure 8b). This extension is made uniformly within V2 and V3. We hence shifted the pacman grid by the amount of a new parameter, ***λ***. In particular, the entire V1 will be shifted to the left by ***λ***. For V2 and V3 - that is for |*θ*| greater than π/2 - the amount of shift is graded. Consider the positions of the intermediate pacman step given in polar coordinates (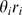) then the amount of shift 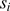 is given by:
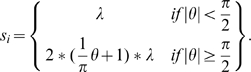
(8)As the intermediate stage is shifted by 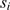 in Cartesian space, the shifted positions in polar coordinates 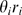 given by
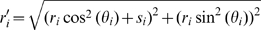
(9)and
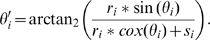
(10)Most of the complexity of these equations arises from transforming the mapping from polar coordinates to Cartesian space and back. This is because the shift is most easily conceptualized in Cartesian space, while both the original *θ_i_*, *r_i_* as well as the shifted coordinates *θ_i_′*, *r_i_*′are in polar space. Whilst the algebraic form looks complicated, the actual computational implementation in a high level language such as Matlab consists of 3 simple lines (see Protocol S1y for example code).

**Figure 9:**
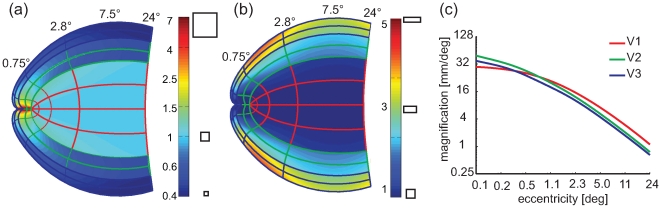
Analysis of the Banded Double-Sech model. (a) The predicted meridional anisotropy is similar to the Double-Sech model, except for the occurrence of a large increase in magnification in the central foveal projection of V2 and V3. (b) At the same time it reduces the local anisotropy in V2 and particularly in V3 compared the original Double-Sech model. (c) The predicted magnification is consistent with empirical reports, with larger magnification in the very foveal portion of V2 and V3.

Figure 9 depicts the predicted anisotropies and magnification from the Banded Double-Sech model. The expanded representation of V2 and V3 in the central fovea results in meridional anisotropy, although serendipitously it reduces the amount of local shear in the range close to the fovea, resulting in an almost locally isotropic representation of all three early visual areas in the very foveal center. With respect to magnification, the Banded Double-Sech model predicts curves for *M_a_* similar to those observed experimentally (compare Figure 9c and 6c). The model introduces one additional parameter, *λ* specifying the amount of shift. We fitted this parameter (*λ* = 0.4) to achieve an optimal prediction of the magnification functions depicted in Figure 6c.

## Discussion

The principal objective of this paper is to propose a parametric map for multiple visual areas, based on recent empirical advances [3]. First, we examined the Wedge-Dipole model [2]. Analysis of the intrinsic features and predictions of this model with realistic parameters suggest that this model is inadequate to describe the architecture reported for the central part of the V1, V2 and V3 complex [3],[15],[26],[27]. Comparing the model with such data, we suggested complementing the Wedge-Dipole concept with a more principled transformation function, the Double-Sech model. With an additional modification to generate the Banded Double-Sech version of this new model, we can now account for all the measured features of the architecture of visual projection areas V1-3. This results in an adequate representation of the V1, V2 and V3 complex from 0 to 16° of eccentricity (Fig. 9), and potentially the full peripheral range.

The novel model we advance hence integrates the results of several retinotopic studies of human early visual cortex [3],[15],[27] providing a full 2D interpretation and interpolation of the available data, and predicting numerous details not explicitly reported by these studies. This endeavor, in turn, provides a ready reference for ongoing research investigating and modeling of the visual stream. We propose that the Banded Double-Sech model can be fitted to retinotopic data of individuals, in a straightforward fashion. Although this manuscript does not investigate the parameter space with respect to inter subject variability, based on our experience with the data at hand [15], we propose that a fairly tight set of parameters, with *a* = 1.05°, *b* = 90°, *λ* = 0.4 will fit the majority of subjects with good accuracy. In other words, the model advanced in this study could be used as anatomical *prior* to allow estimation of inter-areal parcellation of the visual cortex from appropriate – potentially quite sparse - retinotopic data.

Previous work [15],[27] has shown that the scaling parameter *k* will show the greatest variation ranging from 15–26 between subjects. Estimating of *b* is problematic since *b* is mostly constrained by peripheral data. Though there are reconstructions of complete human V1 [28],[29], these reconstructions do not contain retinotopic information. As a result the estimate of *b* is not well constrained and may vary between 40–140°. For a thorough estimate of the complete set of parameters, including the periphery, detailed retinotopic mappings including 2D estimates of the peripheral visual field, i.e. beyond 30° would be required, unfortunately such data currently do not exist.

### Weighting Benefits and Penalties

As we have demonstrated, the retino-cortical projection can be optimized either towards minimal surface area requirements, or towards local isotropy. Comparing these theoretical alternatives with empirical data shows that visual cortex organization is mostly optimized towards minimal surface requirements. While the benefits of saving cortical surface are obvious, the benefits of a locally isotropic representation of the visual environment are more elusive. Neurons in visual cortex are connected to their neighbors, hence processing visual information of their immediate neighborhood. If the retino-cortical projection is locally isotropic, lateral connection networks that are isotropic on cortex would suffice. An anisotropic map, on the other hand, would probably require that that the pattern of horizontal connections counterbalances this anisotropy. That is, local connections would have to be arranged less densely in the direction of maximum relative magnification. Tracer studies show that horizontal connections are very selective, forming series of often elongated patches connecting cells of corresponding properties [25],[30],[31]. The widespread existence of local anisotropy in empirical data suggests that approximating isotropy may probably suffice, exerting a relatively small force in relation to the other constraints of three adjacent maps.

Further, local anisotropies can be compensated by the arrangement of second order mapping features, such as ocular dominance columns, orientation columns or, as suggested for the segregation of V2 into stripes [24]. Effectively, this may result in map fragments that are locally isotropic or even conformal. Computational models suggest that secondary mapping features will be preferentially arranged orthogonal to the anisotropies in a primary map [32]. Accordingly the local anisotropies predicted by our model should constrain secondary mapping features. Thoroughly testing this is beyond the scope of this manuscript, but we would like to point out that the pattern of ocular dominance columns reported agrees well with this notion and matches the prediction of the Double-Sech model [28],[33],[34] in being aligned orthogonal to the vertical meridian map along the boundary of V1, but being poorly organized in the center of the map, where the model predicts minimal anisotropy.

### The Dipole Parameter b and the Periphery

While it is clear that the Monopole model is insufficient to model either the complete V1 or the V1-3 complex in general, the relevance of the peripheral pole described by *b* in the dipole models is naturally for peripheral data. To date there is no sufficiently precise peripheral 2D data, particularly for humans, to obtain accurate estimates of *b*. However, we previously demonstrated that incorporating a fixed *b* = *90* significantly improved the accuracy of the fits even for central data [15]. For reasons of consistency and completeness, throughout this paper we have employed the Dipole model [20], rather than a Monopole model. We used a fixed value of *b* = 90°, consistent with previously suggested values for *b* ranging from 85° to 180° [2],[35].

### Curved Surface, Intrinsic Curvature

The Banded Double-Sech model that we propose here is a strictly planar, two-dimensional one; it does not embody any possibility of curvature in the third dimension. It has been shown for human [15] that a flat 2D model is not only sufficient, but accurately predicts certain features that simple curved surface models would not. Accordingly, a curved surface model for parafoveal human V1 is not only unnecessary, but incompatible with the empirical results. However, while this is valid for parafoveal V1, it may not be correct for the full complex of V1, V2 and V3 and may further not be true in detail even for the central fovea. Unfortunately, there are currently no data available for informing the critical aspects of such models. The data that have been published are too sparse to test if models with a curved surface improve the fidelity of the model. It has to be considered that the relevance of intrinsic curvature increases with the ratio of the modeled part of cortex to the absolute amount of cortex. The relative amount of cortex V1 occupies increases for smaller primates (and accordingly also V2 and V3), and intrinsic curvature would arguably be more significant for a map in these species than in human [26],[36]. We suggest that further improvements of the retino-cortical projection functions may consider curved surface implementations, but at this stage the available data are insufficient to constrain such a model.

### The Effect of the Banding

The banding architecture has two major effects on the cortical maps of V2 and V3. First, consistent with empirical data, it predicts a meridional anisotropy in the central fovea (Figure 9a). In other words the Banded Double-Sech model results in an increased surface area, *i.e.* more neuronal substrate for the foveal representation of V2 and V3 in comparison to V1. The pattern of meridional anisotropy suggested by the Banded Double-Sech model is opposite to that of the classical Log-Polar transform (compare Figures 4a and 9a).

A second prediction of the Banded Double-Sech model, going beyond the precision of the available data, is local isotropy for the central 0.5°. Although, as argued above, local wiring may sufficiently compensate for local anisotropy, it is nonetheless probably a desirable property, particularly for central vision where the functional role of lateral inhibition may be most pronounced [37]. In short, the banded architecture results in two positive effects for the cortical representation of the fovea – increased neuronal substrate for V2 and V3, and decreased local anisotropy. Evolutionary pressure to optimize each of these - alone or in combination - may explain the presence of the banded architecture in human cortex. We suggest that this proposal could be tested by defining cost functions on each of these distortions and implementing an iterative genetic algorithm [38] interpreting the putative computational costs of such distortions in the human cortex.

### Computational Implementation of the Banding

We have suggested a simple procedure to integrate the measured banding of the cortical representation into existing two-stage models, consistent with the enhanced magnification central to 0.5° eccentricity for V2 and V3 relative to V1. The exact implementation for the banding will no doubt need to be further refined as more accurate data become available. The modeled magnifications in Figure 9c suggest that V2 and V3 have slightly different magnifications in the fovea, whereas the empirical data suggest they have essentially the same magnification. One could easily modify the banding function to match these results. However, doing so would risk over-fitting the precision of the model to noisy data [3].

The banding as implemented in the Banded Double-Sech model projects a point (the very center of the visual field) onto a line in V2 and V3. We understand this feature as the special highly localized extreme of the systematic anisotropies in the visual cortex map. One morphogenetic strategy for ameliorating this extreme distortion is by the arrangement of a secondary map along this line, such as orientation preference. While there are currently no data supporting this suggested architecture, we would suggest this as a key feature that could be investigated informing our understanding of the mapping principles on cortex.

### Level of Knowledge, Future Work

The set of equations underlying the Banded Double-Sech model provide an explicit, parametric model of the retinocortical projection for the complex of early visual areas V1–3 from 12° eccentricity down to the central fovea (roughly two thirds of the area of these cortical maps). This model is accurate to the level of our knowledge about the actual organization of these areas in human and possibly other primate species. In particular, there is a paucity of studies in humans and primates of the layout of the foveal confluence for eccentricities below 1°. The principal layout has only recently been reported in humans [3], but is still a matter of debate in macaque [26],[39]. At this stage the model can inform the collection of data by providing detailed predictions to be tested, ideally using high-resolution optical imaging techniques. For humans, the distinct pattern of anisotropies predicted by this model can be further tested using psychophysical performance measures [7],[9]. Imaging studies can use the model to predict the cortical layout of early visual areas in the foveal center, where the retinotopic maps are notoriously hard to obtain [40],[41],[42],[43].

### Conclusions

We have demonstrated that the complex of early visual areas V1, V2 and V3 in primate visual cortex can be optimized towards either space savings or local isotropy, but not both. A new model closely informed by empirical data suggests that the representation of the periphery is optimized for conservation of cortical surface, while the central fovea is locally isotropic. This demonstrates that the retino-cortical projection follows clear morphogenetic principles.

## Materials and Methods

The empirical data of this manuscript are predominantly derived from previous studies [3],[15],[27]. The modeling and results presented here were implemented in Matlab 7.6 (Mathworks, MA). We provide the code in the Protocol S1 of Supplementary Materials, enabling concrete and unambiguous specification of the computing methods employed, and the possibility to further explore the parameter space. This computation was chosen to closely mimic procedures from empirical work [3],[15],[27].

To test local and meridional anisotropy a finely meshed grid in the visual field was projected through the models. Squares of the grid were oriented in such a way that one side was orthogonal to eccentricity, while the other side was orthogonal to polar direction. In principle, anisotropies can be derived analytically [15], however the computational approach implemented for this manuscript allows flexible and comparable testing of model variations. Since we provide the code, the reader can easily implement alternative model functions within the code and test these using the methods provided.

Local anisotropy for a given position in the projection was then calculated as the length ratio of the side oriented parallel to isoeccentricity lines (i.e. *M_e_*) divided by the length of the side parallel to isopolar lines (*M_p_*).
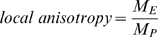
(11)Meridional anisotropy is calculated based on the surface area of a set of squares with the same eccentricity, but varying polar position. Meridional anisotropy for a given position in the projection was then calculated as the surface of a square at this position (*M_a_(P,E′)*) divided by the surface of a square at the horizontal meridian in V1 (*M_a_(0,E′)*).
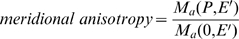
(12)Predicted areal magnification *M* (Figure 6c, Figure 9c) was estimated by projecting isoeccentricity bands. Areal magnification is then the square root of the projected surface divided by the surface in visual space.
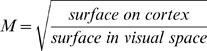
(13)Analytical considerations [15], have shown that this estimate of *M* is the most informative.

## Supporting Information

Protocol S1 Matlab code demonstrating the model.
(0.02 MB ZIP)
